# Spermidine-induced improvements in water relations and antioxidant defense enhance drought tolerance in yarrow (*Achillea millefolium* L.)

**DOI:** 10.1016/j.heliyon.2024.e41482

**Published:** 2024-12-25

**Authors:** Sajedeh Alijani, Mohammad-Reza Raji, Zohreh Emami Bistgani, Abdollah Ehtesham Nia, Mostafa Farajpour

**Affiliations:** a Department of Horticulture, College of Agriculture, Lorestan University, Khoram Abad, 44316-68151, Iran; b Isfahan Agricultural and Natural Resources Research and Education Center, Agricultural Research Education and Extension Organization (AREEO), Isfahan, 81748-35117, Iran; c Crop and Horticultural Science Research Department, Mazandaran Agricultural and Natural Resources Research and Education Center, AREEO, Sari, Iran

**Keywords:** Adaptation, Biochemical pathways, Chlorophyll, Flavonoids, Polyamines

## Abstract

Drought stress poses a serious threat to agricultural productivity worldwide. This study investigated the mitigative effects of exogenous spermidine on drought stressed yarrow (*Achillea millefolium* L.). Plants were subjected to three drought levels (25 %, 50 % and 75 % field capacity) and foliar sprayed with 0, 1.5 and 3 μM spermidine. Drought significantly reduced relative water content, photosynthetic pigments (chlorophyll, carotenoids), osmolyte (proline, soluble sugars) accumulation and antioxidant enzyme activities such as catalase (CAT), peroxidase (POD) and ascorbate peroxidase (APX), indicating oxidative damage. Spermidine treatment attenuated drought injury by improving the above parameters. Maximum responses were observed at 1.5 μM for photosynthetic pigments and osmolytes, while 3 μM performed best for secondary metabolites (phenolics, flavonoids, anthocyanins) and antioxidant enzymes. Drought also upregulated secondary metabolites like phenolics, while spermidine further augmented their levels. Moreover, spermidine maintained membrane integrity and osmotic adjustment under water deficit. Overall, spermidine enhanced yarrow's drought tolerance by modulating physiological and biochemical processes. Our findings provide insights into spermidine-induced adaptation mechanisms in plants combating water scarcity. Optimization of spermidine concentration may help develop drought-resilient crops.

## 1 Introduction

*Achillea* L. is an important medicinal plant of the Asteraceae family, recognized globally for its therapeutic applications [1]. Various species of *Achillea* have been traditionally used to treat ailments such as wounds, headaches, and inflammation [2]. Phytochemical analyses have identified a range of bioactive compounds in *Achillea* spp., including terpenoids, flavonoids, and phenolic acids, which likely contribute to their medicinal properties [3]. The historical use of *Achillea* is well-documented in traditional medicine systems, and modern studies support its efficacy through a rich phytochemical profile and pharmacological activities [4].

Common yarrow (*Achillea millefolium* L.), a perennial herb, typically grows 20–90 cm tall and features delicate leaves and hairy stalks [5]. It has been traditionally employed to alleviate dysmenorrhea and reduce menstrual bleeding, attributed to its antispasmodic and anti-inflammatory properties [6].

Medicinal and aromatic plants are often resilient in challenging environmental conditions, particularly drought, making them valuable in arid regions [7]. Given the increasing prevalence of drought due to climate change, it is crucial to develop strategies to enhance the drought tolerance of these plants. Drought stress significantly affects the growth and production of medicinal plants, leading to reduced yields and compromised synthesis of valuable secondary metabolites [8,9]. While mild, short-term drought can enhance certain compounds, prolonged water deficits generally diminish the quality and quantity of active ingredients [10]. Plants respond to drought through various morphological, structural, and biochemical adaptations; however, severe drought can compromise these protective mechanisms, leading to decreased photosynthesis and increased oxidative stress [11].

As global food demand rises, ensuring the productivity of medicinal plants in water-limited areas is essential. Strategies that promote resistance to intermittent drought through the selection of stress-adapted genotypes and management practices will support sustainable cultivation.

Spermidine (Spd), a key polyamine in plants, plays a critical role in growth and stress responses. Its endogenous levels increase under drought conditions, contributing to protective mechanisms [[12], [13], [14]]. Spd enhances drought tolerance by regulating stomatal behavior and activating stress defense processes via ABA-mediated gene expression [15,16]. For instance, Spd modulates stomatal aperture to minimize transpirational water loss [17]. Exogenous applications of Spd have been shown to alleviate drought stress in various species, including *Vaccinium corymbosum* [18], *Calendula officinalis* [19], and *Tropaeolum majus* [20], due to its membrane-stabilizing and ROS-scavenging properties.

The plant antioxidant system, crucial for managing reactive oxygen species (ROS) generated during stress, consists of enzymes such as catalase (CAT), ascorbate peroxidase (APX), and polyphenol oxidase (POD), as well as small molecular weight antioxidants like proline [21]. Drought also disrupts osmotic balance, with proline serving as a major osmoprotectant that helps regulate osmotic pressure. Increased proline synthesis can mitigate salinity effects on cellular processes. Additionally, total phenolics, flavonoids, and anthocyanins are important secondary metabolites with antioxidant properties in yarrow [22]. Soluble carbohydrates provide energy and act as protective osmolytes during stress [23]. Photosynthetic pigments, including chlorophyll and carotenoids, are also affected by water deficit, impacting photosynthesis and overall plant growth. Together, these factors reflect the complex defensive and metabolic responses of plants to drought conditions.

In this study, we hypothesized that spermidine (Spd) application can enhance the drought tolerance of *Achillea millefolium* (common yarrow) by improving physiological and biochemical responses under water-stressed conditions. The primary purpose of our research was to investigate the effects of exogenous Spd on various parameters, including antioxidant activity, osmotic regulation, and secondary metabolite synthesis, in yarrow subjected to drought stress. By elucidating these mechanisms, we aimed to provide insights into the potential of spermidine as a management strategy to improve the resilience and productivity of medicinal plants in increasingly arid environments.

## 2 Material and methods

### 2.1 Experimental condition

In the study, a factorial experiment based on a completely randomized design with four replications was carried out in the greenhouse located at the Agricultural Research and Education Center in the province of Isfahan. The yarrow seeds used in the experiment were obtained from the medicinal plants area belonging to the same research facility. The seeds were transplanted into the greenhouse in early December when average daily temperatures ranged from 10 to 15 °C.

Two-week old plants were transplanted into pots containing a standard potting medium (each pot had a diameter and height of 30 cm). The results of soil physical and chemical properties were presented in Table 1. An acclimation period was observed to ensure proper root establishment prior to experimental treatments. During this period, irrigation was administered to maintain soil water content at 50 % of available water holding capacity based on gravimetric measurements, in order to stabilize the cuttings without inducing moisture stress.

**Table 1:** The results of soil physical and chemical properties.

EC (dS m^−1^)	pH	Total neutralizing value	Organic carbon	P (ava)	K (ava)	Fe (ava)	Mn (ava)	Zn (ava)	Cu (ava)
		(%)		(mg kg^−1^)					
1.23	7.7	39	2.3	9.7	643	3.14	12.38	1.13	1.1

A 3 x 3 factorial design was employed, consisting of two factors: (1) water stress levels at 75 % (well-watered control), 50 %, and 25 % of available soil water content, and (2) spermidine concentrations at 0 μM (control), 1.5 μM, and 3 μM.

The potting medium had a water holding capacity of 18 % (w/w) as determined through moisture release curves. Drought stress was applied using a weight-based method. The field capacity of the potting soil was determined to be 18 %. The average weight of the pots was recorded at 8 kg. Based on this weight method, the pots experiencing drought stress were irrigated with 75 %, 50 %, and 25 % of the available water, corresponding to weights of 7 kg, 6.5 kg, and 6 kg, respectively. The average irrigation intervals were established as follows: every 3 days for 75 % available water, every 7 days for 50 % available water, and every 10 days for 25 % available water [24].

Prior to water stress initiation, spermidine was exogenously applied through foliar spraying at 2-week intervals, continued throughout the experiment.

### 2.2 Leaf relative water content (RWC)

Leaf relative water content (RWC) was determined by selecting fully developed leaves from plants under different treatment conditions. The leaves were cut into 1-cm pieces and the fresh weight of harvested leaf samples was immediately recorded. The samples were then soaked in distilled water at 25 °C for 16 h, after which their weight was measured again. The samples were dried in paper bags in an oven at 70 °C for 48 h to obtain the dry weight. RWC was calculated using the formula: RWC = (fresh weight - dry weight)/(turgid weight - dry weight) x 100, where turgid weight refers to the weight after soaking leaves in distilled water for 16 h [25].

### 2.3 Photosynthetic pigments

The levels of total chlorophyll, chlorophyll *a*, chlorophyll *b*, and carotenoid were determined using a spectrophotometer. The concentrations of pigments were then calculated based on an equation that took into account the absorbance values recorded at 662 nm (A661.6), 645 nm (A644.8), and 470 nm (A470).

Chl a = 11.24 × (A_661.6_) – 2.04 × (A_644.8_)

Chl b = 20.13 × (A_644.8_) – 4.19 × (A_661.6_)

Cartenoide = (1000 × A_470_ – 1.9 × Ch a – 63.14 × Ch b)/214

### 2.4 POD activity

Peroxidase enzyme activity was determined based on the formation of tetraguaiacol from guaiacol in the presence of hydrogen peroxide and enzyme extract. Absorbance was read at 470 nm over 3 min. The amount of tetraguaiacol produced was calculated using the molar extinction coefficient (26.6 mM^−1^cm^−1^). Enzyme activity was expressed as micromoles of tetraguaiacol formed per minute per milligram of sample protein. In the equation used, the change in absorbance between the beginning and end of the reaction (A470Δ) represented U (enzyme unit), while other factors accounted for were the H_2_O_2_ coefficient (4), total reaction volume (3063 μL), dilution factor (1.60), reaction time (300 s), sample volume (50 μL), molar extinction coefficient (6.26 mM^−1^cm^−1^), and path length (1 cm) of the reaction mixture [26].

Activity(Uml)=ΔA240×l×Vt×dfε×l×t×Vs

### 2.5 CAT activity

Catalase enzyme activity was determined at 25 °C using a spectrophotometer set to a wavelength of 240 nm. The reaction mixture contained 3000 μL of 50 mM phosphate buffer at pH 7, 5 μL of 30 % hydrogen peroxide solution, and 50 μL enzyme extract sample. Absorbance readings were taken at 20-s intervals over a period of 5 min to monitor the reaction. Catalase activity was expressed as micromoles of hydrogen peroxide decomposed per minute per milligram of protein based on the change in absorbance over time [27].

### 2.6 APX activity

About 0.1-g leaf sample was homogenized with 1000 μl of buffer containing sodium phosphate and PVP. The solution was then centrifuged at 14,000 rpm for 20 min at 4 °C. An extraction mixture was prepared containing 1500 μl acid-free extraction buffer, 300 μl buffer with ascorbic acid, and 3 μl oxygenated water. This was mixed with 50 μl of the supernatant and read at 290 nm using a spectrophotometer. APX activity was calculated as micromoles per minute per gram fresh weight using an equation [26] that took into account the difference in absorbance at 100 and 40 s (A100-A40) at 240 nm, the total volume in the cuvette (Vt), the ascorbate decomposition coefficient (constant value of 8.2), and the volume of the supernatant (Vs).

E × Vs/[Vt × (A_100_-A_40_)] = APX activity

### 2.7 Proline content

Proline content was determined following the procedure of Bates et al. [28] using a spectrophotometer at 25 °C. Fresh leaf tissue (0.2 g) was homogenized in a mortar with 10 mL of 3 % sulfosalicylic acid. The extract was centrifuged at 13,000 rpm for 10 min at 4 °C. Two mL of supernatant was transferred to tubes, along with 2 mL each of ninhydrin reagent and glacial acetic acid. The tubes were sealed and incubated at 100 °C for 1 h. After cooling, 4 mL of toluene was added and absorbance read at 520 nm. Proline was quantified against a standard curve and expressed as mg per g fresh weight.

### 2.8 Soluble carbohydrates

To extract carbohydrates, 0.2 g leaf tissue was heated with 10 mL 95 % ethanol at 80 °C for 1 h. Then, 1 mL 5 % phenol and 5 mL 98 % sulfuric acid was added. Absorbance at 483 nm was read using a spectrophotometer. Amount of extracted carbohydrates was determined against a glucose standard curve in μg per g fresh weight.

### 2.9 Anthocyanin concentration

Anthocyanin concentration was determined following the procedure described by Wanger [28]. Fresh leaf tissue (0.1 g) was homogenized in 10 mL of acidic methanol using a tissue grinder. The extract was incubated in the dark at 4 °C for 24 h to enhance extraction of pigments. Samples were then centrifuged at 4000 rpm for 10 min to separate solids. Absorbance of the resulting supernatant was measured at 550 nm using a spectrophotometer. Anthocyanin content was calculated using following equation:

c=A/єb

Where: c = Anthocyanin concentration; A = Absorbance; є = Extinction coefficient of 33,000 M^−1^cm^−1^; b = Path length.

### 2.10 Total phenol content

Total phenolic content was determined using the Folin-Ciocalteu colorimetric method [29]. To 10 μL of methanol plant extract, 500 μL of Folin-Ciocalteu reagent was added. Samples were vortexed for 5 min, followed by the addition of 3 mL 1 % sodium carbonate solution to yield a blue colored reaction mixture. After incubating at room temperature for 2 h, absorbance was measured at 760 nm using a Spectromax-M5 spectrophotometer. Gallic acid was used to construct a standard calibration curve, and results for extracts were expressed as milligrams of gallic acid equivalents per gram based on the linear equation. This spectrophotometric assay relies on electron transfer in alkaline conditions between phenolic compounds and phosphomolybdic/phosphotungstic acid present in Folin-Ciocalteu reagent, producing a colored complex with absorbance proportional to total phenol concentration.

### 2.11 Total flavonoid content

Total flavonoid content was determined using the aluminum chloride colorimetric method [29]. To 1 mL plant extract, 2 mL of 2 % aluminum chloride solution was added. Samples were gently mixed followed by addition of 6 mL 5 % potassium acetate solution. The reaction mixture was incubated at room temperature (37 °C) for 40 min to complete color development. Absorbance was then measured at 415 nm using a Spectromax-M5 spectrophotometer. Rutin was used to prepare a standard curve and results for extracts expressed as milligrams of rutin equivalents per gram of dry extract weight.

### 2.12 Statistical analysis

The Shapiro-Wilk normality test results indicated that the data were normally distributed (p > 0.05). Since the assumption of normality was met, an analysis of variance (ANOVA) was performed on the data using SAS software v.9.1. The mean values of the data were compared using the least significant difference (LSD) test at a 5 % significance level, and the graphs were plotted using Excel software.

## 3 Results

### 3.1 RWC

**Fig. 1:**
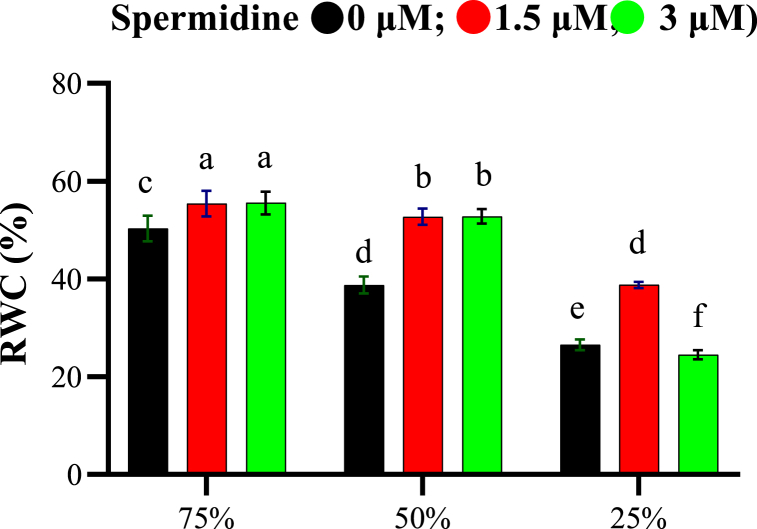
Interaction effects of spermidine on RWC of yarrow under drought stress, analyzed using the LSD test (p < 0.05).

The statistical analysis of variance (ANOVA) showed that drought stress, spermidine treatment, and their combined effect had a significant impact on RWC at the 1 % probability level, as presented in Table 2. RWC showed a decreasing trend with increase in drought stress severity from 75 % to 25 % field capacity (Fig. 1). Under normal irrigation (75 % field capacity), RWC was 50.33 % without spermidine treatment which increased to 55.42 % and 55.56 % with 1.5 and 3 μM spermidine, respectively. A similar increase in RWC was observed under moderate (50 % field capacity) and severe (25 % field capacity) stress on application of spermidine. The maximum protection of 38.78 % RWC was afforded by 1.5 μM spermidine under severe stress compared to 26.51 % without spermidine treatment. The results demonstrated that drought stress significantly reduced RWC while exogenous spermidine ameliorated this effect. Spermidine thus helped maintain higher cellular water content and mitigated the impact of drought on yarrow plants.

**Table 2:** Analysis of variance of different characters of yarrow plants treated by spermidine under drought stress.

S.O.V	df	RWC	Chl a	Chl b	Car	Proline	Carb	ANT	APX	POD	CAT	TP	TF
Drought	2	1395.42∗∗	312.4∗∗	85.03∗∗	34.08∗∗	0.05∗∗	0.3705∗∗	0.19∗∗	0.42∗∗	0.22∗∗	0.8∗∗	16.7∗∗	0.31∗∗
Spermidine	2	246.05∗∗	398.6∗∗	205.48∗∗	26.84∗∗	0.003∗∗	0.0568∗∗	0.027∗∗	0.31∗∗	0.01∗∗	0.46∗∗	1.74∗∗	0.02∗∗
Dro∗Sper	4	77.98∗∗	45.02∗∗	11.17∗∗	5.41∗∗	0.001∗∗	0.0025∗	0.004∗∗	0.21∗∗	0.28∗∗	1.44∗∗	0.11∗∗	0.00∗∗
Error	18	0.98	0.142	0.051	0.0448	0.0003	0.000629	0.0008	0.03	0.00	0.00	0.09	0.00
C.V. (%)	–	2.14	3.02	2.71	5.92	6.03	7.5	8.77	4.17	6.51	4.17	1.87	3.43

RWC: Leaf relative water content; Chl a: chlorophyll *a*; Chl b: chlorophyll *b*; Car: Carotenoid; Carb: Carbohydrate; ANT: Anthocyanin; TP: Total phenol; TF: Total flavonoids.

### 3.2 Photosynthetic pigments

**Fig. 2:**
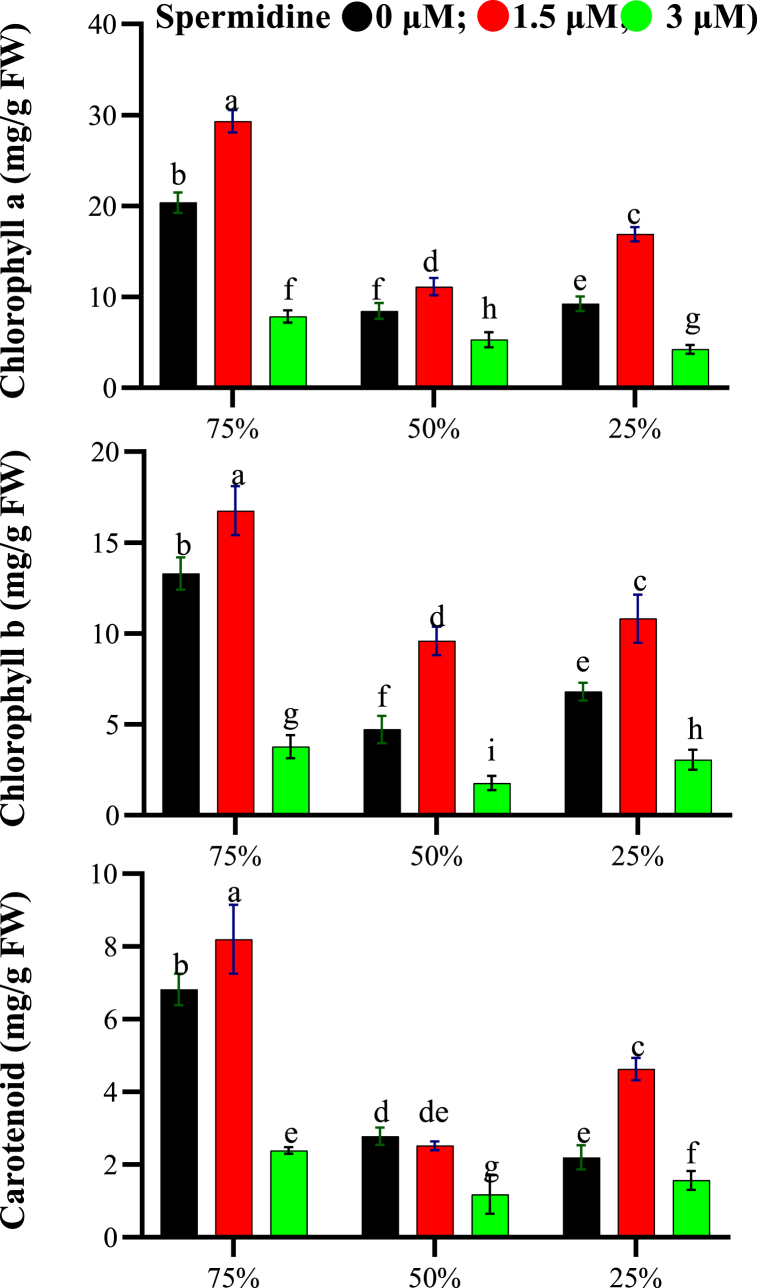
Interaction effects of spermidine on photosynthetic pigments of yarrow under drought stress, analyzed using the LSD test (p < 0.05).

The ANOVA revealed that drought stress, spermidine treatment, and their interaction had a highly significant impact on chlorophyll *a*, *b* and carotenoid content (p < 0.01). These photosynthetic pigments exhibited variation with changes in water stress levels and spermidine concentrations (Fig. 2). Under normal irrigation (75 % field capacity), the highest pigment values were observed with 1.5 μM spermidine treatment - chlorophyll *a* (29.31 mg/g FW), chlorophyll *b* (16.76 mg/g FW), and carotenoids (8.2 mg/g FW). Without spermidine, the pigment levels declined with increasing drought intensity. Even under severe stress (25 %), 1.5 μM spermidine improved pigment content over the control. Based on these results, 1.5 μM spermidine application under all stress levels was most effective for mitigating the deleterious effects of drought on photosynthetic pigments, as evidenced by consistently higher pigment retention compared to 0 and 3 μM spermidine treatments.

### 3.3 Secondary metabolites

**Fig. 3:**
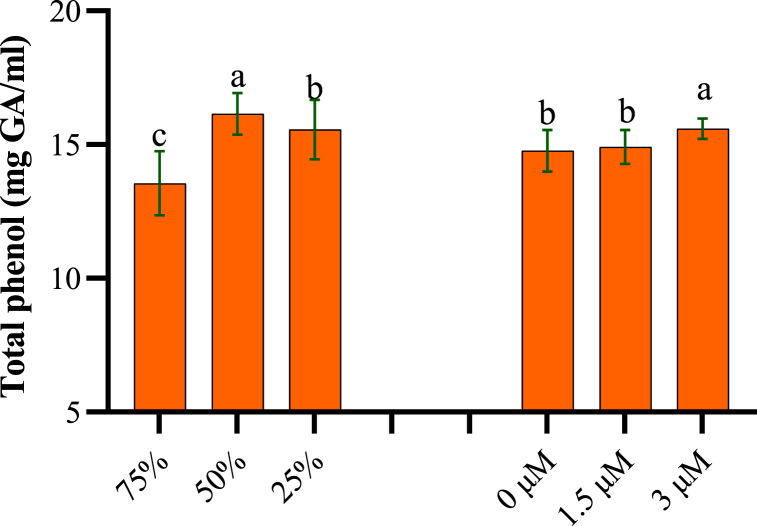
Effects of spermidine and drought stress on total phenol of yarrow, analyzed using the LSD test (p < 0.05).

**Fig. 4:**
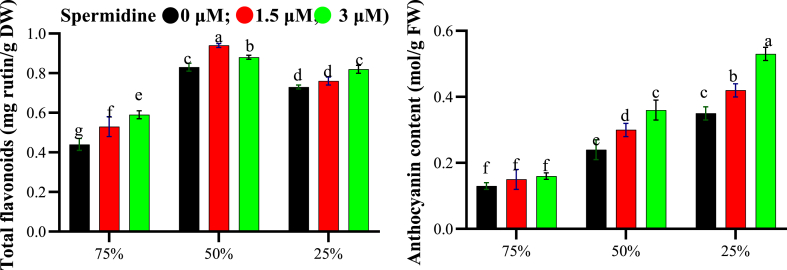
Interaction effects of spermidine on secondary metabolites of yarrow under drought stress, analyzed using the LSD test (p < 0.05).

The results showed the effects of water stress, spermidine treatment and their interaction on total phenols, flavonoids and anthocyanins in yarrow plants. Total phenol content was influenced individually by drought and spermidine, though their interaction was insignificant. The highest phenols (16.15 mg/g FW) occurred at 50 % stress without spermidine (Fig. 3). Severe drought induced 15.56 mg/g FW phenolics accumulation. Spermidine enhanced phenols to a maximum of 15.59 mg/g FW at 3 μM spermidine. Total flavonoid and anthocyanin contents were significantly impacted by stress, spermidine and their interaction. The maximum flavonoid content (0.94 mg rutin/g DW) was recorded at 50 % stress with 1.5 μM spermidine (Fig. 4). Even under severe drought, this spermidine dose elevated flavonoids to 0.76 mg rutin/g DW. Combined drought and 1.5 μM spermidine strongly induced flavonoid biosynthesis. Anthocyanins accumulated more with increasing drought. The peak value (0.53 mol/g FW) occurred under severe stress aided by 3 μM spermidine. This spermidine concentration boosted anthocyanins the most, even at 50 % drought. Combined action of drought and spermidine synergistically promoted anthocyanin accumulation, likely strengthening antioxidant protection under water deficit.

### 3.4 Osmoprotectants

**Fig. 5:**
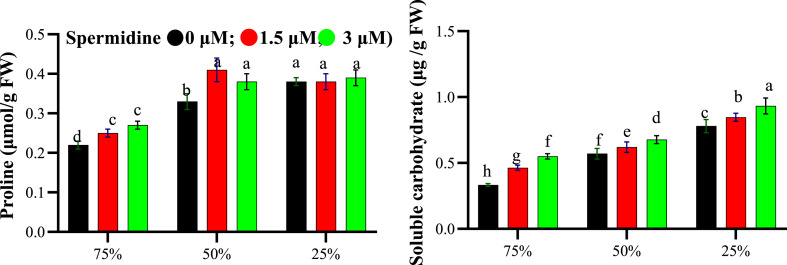
Interaction effects of spermidine on osmoprotectants of yarrow under drought stress, analyzed using the LSD test (p < 0.05).

Proline content varied with the studied factors, with the lowest level (0.22 μmol/g FW) seen at 75 % FC without spermidine (Fig. 5). Under normal irrigation, 1.5 μM spermidine slightly increased proline to 0.25 μmol/g FW. Drought led to proline accumulation, reaching 0.33 μmol/g FW at 50 % stress in control plants. However, maximum proline (0.41 μmol/g FW) was observed at 1.5 μM spermidine under moderate stress. The highest proline under severe stress was also with 1.5 μM spermidine (0.38 μmol/g FW). Soluble carbohydrates followed a similar trend, being highest at 0.933 μg/g FW with severe stress (25 % FC) and 3 μM spermidine. At 50 % FC, this spermidine dose achieved 0.677 μg/g FW carbohydrates. Spermidine application alone elevated carbohydrates over control levels. Drought combined with 3 μM spermidine strongly promoted carbohydrate synthesis, indicating their collaborative role in stimulating osmolytes crucial for cell turgor maintenance amid water scarcity.

### 3.5 Antioxidant enzymes

**Fig. 6:**
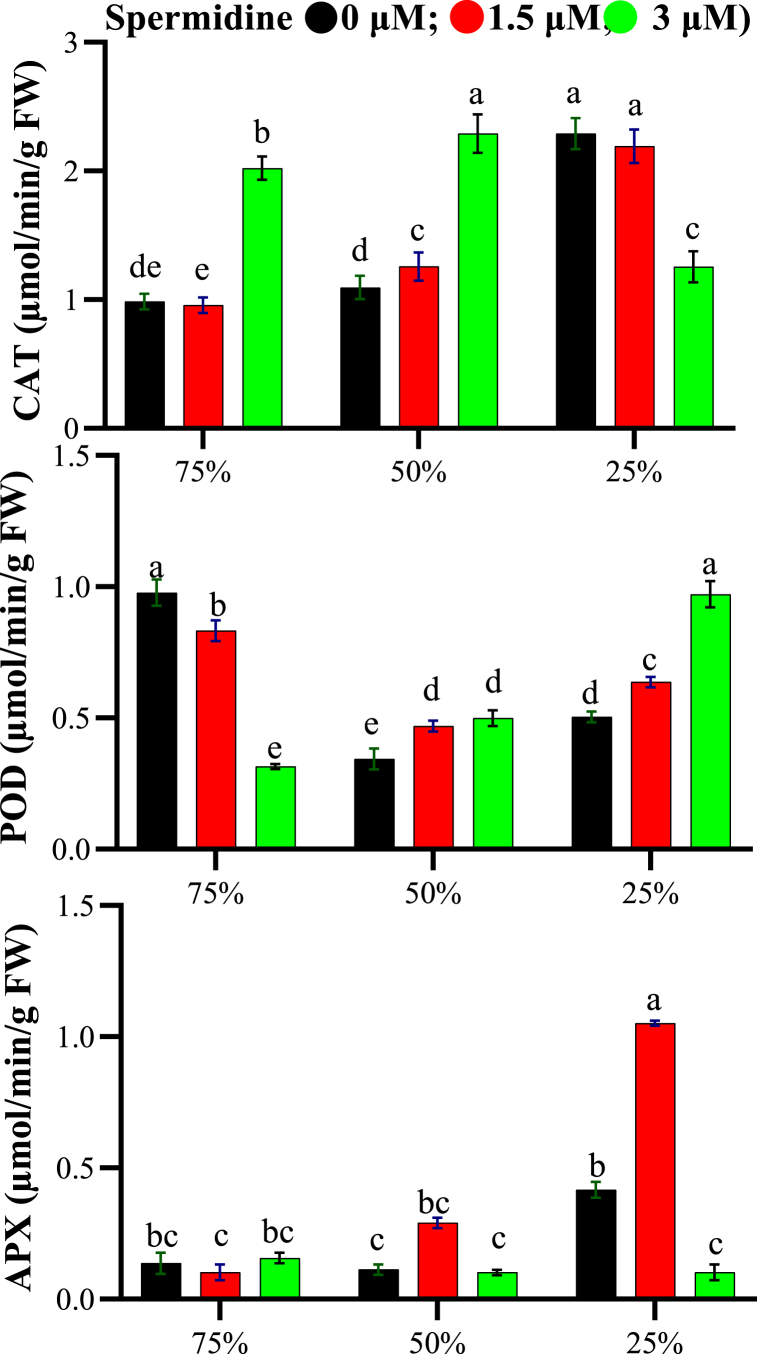
Interaction effects of spermidine on antioxidant enzymes of yarrow under drought stress, analyzed using the LSD test (p < 0.05).

The ANOVA revealed that drought stress, spermidine concentration, and their interaction significantly impacted CAT, POD and APX activity (p < 0.01). For CAT, activity was lowest under control conditions at 75 % field capacity but increased with rising drought intensity, reaching a maximum of 2.29 μmol/min/g FW without spermidine at 25 % FC (Fig. 6). Spermidine application generally boosted CAT levels versus respective stress treatments, with 3 μM spermidine showing highest activity of 2.29 and 2.021 μmol/min/g FW at 50 % and 75 % FC respectively. Meanwhile, 1.5 μM spermidine induced maximum CAT (2.19 μmol/min/g FW) at 25 % FC. Under no spermidine, POD peaked at 0.98 μmol/min/g FW under 75 % FC stress. At 1.5 μM spermidine, POD increased across all stresses compared to controls, with 3 μM spermidine giving highest POD (0.971 μmol/min/g FW) under severe (25 % FC) drought. Regarding APX, activity was lowest (0.113 μmol/min/g FW) without spermidine at 50 % FC but highest (0.42 μmol/min/g FW) under 25 % FC stress. Spermidine generally promoted APX versus respective stresses; maximal APX (1.05 μmol/min/g FW) occurred at 25 % FC with 1.5 μM spermidine. Additionally, 3 μM spermidine yielded highest APX (0.16 μmol/min/g FW) under 75 % FC non-stress. Overall, drought stress and spermidine synergistically enhanced antioxidant enzyme activity in yarrow leaves.

## 4 Discussion

According to the results of present study drought stress was found to decrease RWC, whereas spermidine application increased RWC. Higher leaf RWC confers greater ability to maintain water in plants. Moreover, RWC exhibits high correlation with leaf water potential. Decreased RWC leads to stomatal closure, reduced photosynthesis, and at severe levels can cause electron transfer inhibition, photoinhibition and membrane damage. It is plausible that polyamine application acts as a compatible osmolyte, mitigating the reduction of RWC under stress conditions by reducing osmotic stress and sustaining water uptake [30].

The present study showed that at 50 and 25 % field capacity, photosynthetic activity was inhibited and chlorophyll content changed. Under drought, photosynthesis declined due to stomatal closure. The decrease in chlorophyll pigment content under stress may be attributed to increased degradation or decreased production of these pigments, and disruption of enzyme activity regulating photosynthetic pigment synthesis [31]. Moreover, specific membrane protein reduction under stress and elevated chlorophyllase and peroxidase enzyme activity can influence chlorophyll decline under stress [32]. Glutamate allocation for proline synthesis, a common precursor for proline and chlorophyll involved less in chlorophyll production for coping with osmotic stress [33]. Carotenoids act as accessory light-harvesting pigments but their antioxidant capacity mitigates various activated oxygen forms generated via photosensitization of light-produced photosynthetic compounds [32]. Carotenoids absorb energy from excited oxygen and convert it to heat. Though less stress-sensitive than chlorophylls, water and salinity stress decreased carotenoids in some plants [32]. Spermidine application at 1.5 μM increased photosynthetic pigment content based on results. Previous reports indicate chlorophylls and carotenoids decrease under environmental stress or senescence, whereas polyamines reduce chlorophyll degradation and improve light absorption for enhanced photosynthesis, though their mechanism remains unclear [[34], [35], [36]]. Polyamine-mediated chlorophyll increase may relate to their antioxidant properties preventing chloroplastic membrane degradation [37].

This study investigated the effect of drought stress on proline content in yarrow. Results showed proline accumulation under drought, with the highest levels at 25 % field capacity indicating stress resistance [38]. Proline increase is attributed to its roles in regulating and protecting osmosis, whereby greater proline enhances tolerance to osmotic stresses. Reduced root-zone water potential along with declining leaf relative water content and intensified drought/salinity induced lipid peroxidation and disrupted membrane function/structure, activating glutamine kinase - the first enzyme in proline biosynthesis - thereby elevating proline levels [39]. Cytoplasmic proline accumulation helps maintain water potential balance while imparting least growth inhibition versus other amino acids under oxidative attack [40]. This justifies proline's stress-mitigating role, especially in drought and osmosis regulation. Under drought, proline maintains osmotic potential, scavenges reactive oxygen species and radicals, guards macromolecules from denaturation and regulates cellular pH [41]. Spermidine increased proline consistent with previous findings [42]. Polyamines may exhibit pleiotropic effects through antioxidant promotion and free radical elimination plus influence on enzymatic systems like those regulating proline [42].

In this study, carbohydrates increased significantly with rising drought levels, with maximum levels at 25 % field capacity under 3 μM spermidine application. Certain carbohydrates are reported to mitigate drought inhibition of photosynthesis gene transcription; for instance, drought stress expression of genes encoding Rubisco subunits indicates a controlled regulatory mechanism potentially explaining leaf carbohydrate accumulation [43]. Additionally, this rise may reflect the ability of tolerant genotypes to maintain stomatal aperture and sustain photosynthesis under drought. Drought tolerant plants withstand water deficit by accumulating protective osmolytes like carbohydrates and proline to balance cellular osmotic potential without hindering metabolism. Upregulating biosynthetic pathways and preserving biochemical functions permits continued carbon assimilation and biomass production under limited water availability. Spermidine likely facilitates compatible solute accumulation and membrane/protein integrity, further enhancing the drought response [44]. Maintaining metabolic processes through osmotic adjustment is critical for drought tolerance in plants facing water scarcity.

This study investigated the effects of drought stress and spermidine on anthocyanin, phenolic, and flavonoid compounds. Maximum anthocyanins occurred under 25 % drought stress and 3 μM spermidine treatment. Anthocyanins are water-soluble flavonoids synthesized in the flavonoid pathway [45]. Oxidative stress induces antioxidant gene and phenylpropanoid pathway expression, especially flavonoid biosynthesis. Drought upregulates phenylalanine ammonia lyase, a key enzyme in flavonoid/anthocyanin biosynthesis, thereby elevating compound levels [46]. Phenolic accumulation is a non-enzymatic drought-induced oxidative stress defense [47]. In this study, spermidine (3 μM) increased yarrow phenols, which can be potentially by inhibiting IAA oxidase and promoting auxin/growth under stress defense [47]. Polyamine-treated stressed yarrow exhibited higher aerial organ phenolics, perhaps due to enhanced biosynthesis of beneficial compounds [48]. Spermidine (1.5 μM) elevated flavonoids at 50 % drought, and 3 μM significantly increased flavonoids at 25 % drought. Stress-induced phenolic accumulation may activate cascades enhancing tolerance [49]. While polyamine functions are proposed, their role in modulating phenolic metabolism is unexplored. Polyamines could alter stress tolerance through impacts on phenolic metabolism, synergism or accumulation [50].

The results of present study showed that CAT and APX activity rose significantly with increasing drought levels. Drought and other abiotic stresses induce oxidative stress. Ascorbate peroxidase activity elevates under stress due to heightened reactive oxygen species activating signaling pathways enhancing antioxidant enzyme gene expression and enzyme activity [51]. Antioxidants play a vital role in cellular redox homeostasis by directly reacting with and scavenging various reactive oxygen forms [52]. Decreased activity of some antioxidant enzymes is offset by upregulation of others. Some researchers posit augmented antioxidants constitute the primary stress resistance mechanism, as boosted antioxidant enzyme activity involved in defenses mitigates membrane, DNA and protein oxidation damage [53]. It can be inferred drought increased POD compound levels, followed by accelerated antioxidant enzyme activity and concentration under water deficit to counter radicals [54].

Based on results of current study, spermidine significantly impacted catalase, peroxidase, and ascorbate peroxidase antioxidant enzyme activity under drought stress. Exogenous spermidine was reported to increase catalase activity, protecting cells from free radicals and stresses [55,56]. Catalase and peroxidases are integral to detoxifying hydrogen peroxide by converting it to water and oxygen, preventing cellular damage under adverse conditions like drought [57]. Polyamine antioxidant properties mainly relate to their cationic state at physiological pH, improving ion balance for scavenging free radicals and inhibiting lipid peroxidation [58]. Free polyamine binding to macromolecules confers protection against oxidative damage, while free polyamines primarily regulate cellular osmosis and pH (Zhang et al., 2018). Some polyamine characteristics mimic antioxidants regarding membrane stability and lowering peroxide from changes to polyamine biosynthetic gene expression and consequent biosynthetic polyamine levels [59]. Abundant polyamine production may result from interactions as free forms with anionic molecules like DNA, RNA, proteins and lipid bilayers, unconventionally stabilizing membrane systems under stress (Mustafavi et al., 2016). Research demonstrated spermidine significantly increased CAT, POD, and APX activity in the lettuce under high-temperature [60]. This study provides mechanistic insight into polyamine-mediated enhancement of drought tolerance in plants.

## 5 Conclusion

This study examined the impact of drought stress and exogenous spermidine application on yarrow plants. Drought stress led to reduced relative water content (RWC), photosynthetic pigments, and osmoprotectants. However, spermidine treatment effectively mitigated these negative effects. Both 1.5 μM and 3 μM concentrations enhanced RWC, chlorophyll and carotenoid levels, and osmolytes like proline and soluble carbohydrates under drought conditions, with 1.5 μM being particularly effective for photosynthetic pigments and proline. Additionally, spermidine increased the activity of antioxidant enzymes (CAT, POD, APX) compared to drought alone and boosted the accumulation of beneficial secondary metabolites, such as phenolics, flavonoids, and anthocyanins, with 3 μM showing the greatest effect. Overall, spermidine enhanced yarrow's drought tolerance by modulating physiological and biochemical pathways. The findings suggest that a spermidine concentration range of 1.5–3 μM is optimal for improving drought response mechanisms. Future research should focus on optimizing spermidine concentrations to maximize drought tolerance in yarrow, contributing to strategies for enhancing crop resilience to water scarcity.

## CRediT authorship contribution statement

**Sajedeh Alijani:** Writing – original draft, Methodology. **Mohammad-Reza Raji:** Writing – original draft, Supervision, Methodology, Conceptualization. **Zohreh Emami Bistgani:** Supervision, Methodology, Conceptualization. **Abdollah Ehtesham Nia:** Supervision, Methodology. **Mostafa Farajpour:** Writing – review & editing, Writing – original draft, Supervision, Methodology, Formal analysis.

## Ethics approval and consent to participate

The article does not contain any studies with human participants or animal subjects.

Data and code availability statement.

All data are within the article.

## Declaration of competing interest

The authors declare that they have no known competing financial interests or personal relationships that could have appeared to influence the work reported in this paper.
